# Preferences of psychotherapists for blended care in Germany: a discrete choice experiment

**DOI:** 10.1186/s12888-022-03765-x

**Published:** 2022-02-12

**Authors:** Elena A. Phillips, Sebastian Himmler, Jonas Schreyögg

**Affiliations:** 1 Hamburg Center for Health Economics, University of Hamburg, Esplanade 36, 20354 Hamburg, Germany; 2 Erasmus School of Health Policy & Management Health Economics, Burg. Oudlaan 50, 3062 Rotterdam, PA Netherlands

**Keywords:** E-mental health, Blended care, E-therapy, Interventions, Discrete choice experiment, Therapist preferences, Therapeutic alliance

## Abstract

**Objectives:**

Digital treatment formats are emerging within mental health care. Evidence suggests that mental health care providers and recipients prefer a combination of digital and traditional elements within psychotherapy treatment formats, also called blended care (BC), over standalone digital formats. We examined the attitudes and preferences of licensed psychotherapists in Germany regarding such BC applications.

**Methods:**

We fielded a survey among psychotherapists, including questions about attitudes, previous experiences, and expectations regarding BC, as well as a discrete choice experiment. Attributes for the experiment were developed using a stepwise qualitative approach. A Bayesian D-efficient design was used to generate the choice tasks. The choice data were analyzed by applying mixed logit models.

**Results:**

The survey was completed by 200 psychotherapists. Attitudes towards BC were mainly positive, with strong reported intentions to use BC formats. In the choice experiment, recommendation from a professional society for a BC online component was the most important characteristic. Greater effectiveness and a larger share of face-to-face vs. online time were also desired features, while a financial incentive to use BC was less relevant.

**Supplementary Information:**

The online version contains supplementary material available at 10.1186/s12888-022-03765-x.

## Introduction

In light of the ongoing COVID-19 pandemic, calls for the use of digital technology in mental health have increased [1]. Lockdowns have forced many therapists and patients to turn to videoconferencing as a substitution for face-to-face sessions and rekindled interest in e-mental health interventions, also called online- or web-based therapeutic interventions (eMHIs) [1]. eMHIs are self-help-based, usually short-term therapeutic programs mainly based on cognitive behavior therapy (CBT) for patients with mild and moderate symptoms for a specific psychological condition [2]. eMHIs usually include some remote interaction with a psychologically skilled coach or therapist via e-mail, telephone or video conferencing [3]. eMHIs have been found to be effective in improving mental health and treating psychological conditions, such as psychological distress, burn-out, depression, anxiety, insomnia, eating disorders, or problematic substance use [3–5].

Despite advantages for patients in terms of the flexible delivery of eMHIs, their adherence and acceptability among the general population remain limited compared to face-to-face psychotherapy. Studies on the acceptance barriers associated with eMHIs revealed a strong patient preference for personal contact in the therapy process [6, 7]. Emerging blended care (BC) treatment formats, broadly defined as technology-supported therapy, aims to link the advantages of technology and personal contact with a therapist [8]. Currently, there is no precise definition of BC available, as different types of technology such as eMHIs for a specific psychological condition or online-components with a specific therapeutic functionality or technology that enables secure communication via e-mail or video-conferencing, can be combined with the traditional face-to-face therapy [8, 9]. According to current evidence, patients experience blended formats positively and seem to prefer BC to the standalone use of eMHIs [10–13]. A strong preference for blended types of interventions was also confirmed in a recent study from Germany using a discrete choice experiment (DCE) [11]. Results along the same lines were found in a survey on attitudes towards digital treatment of depression in eight European countries^1^ [12].

The first studies on attitudes towards eMHIs among mental health care providers, predominantly psychotherapists and psychologists, have shown similar tendencies; therapists prefer BC over the standalone use of eMHIs and associate BC with lower risks and disadvantages [13–15]. The first studies have shown that therapists see BC beneficial for a potential improvement in self-management of patients, a more independent patient-therapist relationship, potential better preparation of face-to-face sessions, and time savings on the patient and therapists’ side [8, 13–16]. Attitudes towards BC vary depending on geographical location or therapeutic orientation. Therapists in countries with a higher level of dissemination of e-mental health, such as the Netherlands, Sweden and United Kingdom, express positive attitudes towards BC; by contrast, therapists in countries such as Austria and Germany, with lower e-mental health utilization, or France, with a strong tradition of psychodynamic therapies, emphasize disadvantages and risks regarding eMHIs and hesitation regarding future use [15, 16]. CBT-trained professionals are more positive about eMHIs and blended interventions in general than therapists with other therapeutic backgrounds [14, 15, 17].

In general, while psychotherapists are more open to BC than eMHIs alone, it is still unclear which conditions would encourage the use of BC to integrate technology into therapeutic process. One complicating factor in exploring attitudes towards BC is the lack of coherent understanding or definition of BC, which can be implemented in various forms [8, 16, 18]. The BC format depends on the type of online components used, the extent to which online and personal sessions are combined, and the chronological order in which the online component will be applied—before, parallel to or after personal therapy [8, 13, 18]. Preferences for BC use may depend on the configuration of the BC format and application scenarios; however, no study has investigated the preferences of psychotherapists considering different BC application scenarios thus far.

The aim of our study was two-fold. First, we wanted to explore previous experiences with and expectations for using BC and attitudes towards specific features of BC among psychotherapists in Germany, a country with low e-mental health utilization with an increasing need for timely and adequate psychotherapy [17]. Second, given the low diffusion of BC and hesitation regarding eMHIs among German therapists [15, 17], we were interested in understanding psychotherapists’ preferences regarding BC formats and application modes. Therefore, we conducted a DCE, which entails choices between hypothetical blended treatment options, thus making blended treatment more tangible to participants than is possible with conventional survey techniques. To our knowledge, this is the first DCE that explored preferences among psychotherapists regarding BC and its application scenarios. Knowing which BC application scenarios are preferred by therapists can help policy makers and BC program developers to facilitate conditions and design BC formats that are more attractive to therapists, which could increase the acceptance and uptake of such techniques among different providers in in- and outpatient settings. With citizens and patients in Germany and elsewhere seemingly open to the use of BC [11], this could shift some parts of certain forms of mental health care to a digital format, thus freeing up therapeutic resources for other purposes.

## Methods

We designed a survey with two distinct parts: The first part consisted of a series of questions on therapists’ experiences with and expectations for BC, as well as their attitudes towards specific BC features. To develop these questions, we consulted the related literatureespecially Dijksman et al., who examined the perception and needs of psychologists regarding BC in the Dutch context [16]. We also conducted research to identify further BC online components and inquired about their relevance in exploratory interviews with five psychotherapists. The second part of our survey and study consisted of a DCE, which included four steps: (1) defining attributes and levels for the experiment; (2) generating the experimental design and survey; (3) piloting the survey; and (4) collecting the data. The DCE design development followed best practice guidance, including consideration of the 10-point checklist for conjoint experimental design provided by the International Society for Pharmacoeconomics and Outcomes Research (ISPOR) [19].

### Development of attributes and levels

BC as a technology-supported therapy can be operationalized in various ways depending on the type of technology that is being integrated into the therapy process. We defined an online component as a singular online tool with a specific therapeutic function or an eMHI for a specific psychological condition. Operationalizing BC, we explicitly excluded technology that solely enables remote therapist-patient communication without offering a therapeutic functionality to make the choice alternatives more comparable. Online communication technologies and therapeutic programs or tools represent two distinct functions and therefore in our view should be studied separately. Attributes and levels for the DCE were developed using a stepwise qualitative approach. First, we conducted a literature review on BC with a focus on therapists’ perceptions of and barriers to the use of BC. The literature review was conducted in the PubMed database in July 2020 with “blended care” as main search term. We identified 226 articles on BC with five articles exploring therapist’s attitudes towards BC [8, 13–16]. The main barriers on the side of therapists identified referred to the lack of personal communication, possible negative influence of technology on therapeutic relationship, additional workload associated with BC, uncertainty about effectiveness of technological components, concerns about legal issues/liability, and general uncertainty how to blend technology into the therapeutic process BC [8, 13–16]. In the second step, we structured the identified factors that promote or hinder the use of blended treatment using the unified theory of acceptance and use of technology (UTAUT), formulated by Venkatesh [20]. We have chosen the original version of the UTAUT model due to its better fit for structuring our research questions We believe that the two extensions of the UTAUT2, “habit”, “experience” and “hedonic motivation”, do not (yet) play a significant role for eMHI acceptance in Germany [21]. eMHI technology is at very early stage of adoption in Germany in the moment and no sufficient time has passed to form a habit or identify fun or pleasure in eMHI usage. Venkatesh et al. defined four core determinants of users’ behavioral intention to use a technology: performance expectancy, effort expectancy, social influence, and facilitating conditions [20]. According to previous research, performance expectancy has the largest impact on the intention to use the technology [22]. Tailoring this to the context of BC, we included the attribute *effectiveness* of the online component used in blended treatment in our DCE design. Effort expectancy refers to the perceived difficulty of using the technology. There are different types of online-tools or eMHIs with different technological configurations as well as with a different extent of the involvement from the therapist’s side into the process, which also influences effort expectancy. As the aim of our study was not to find preferences between different types of technology that could be blended, but rather to understand therapists’ preferences for the use of technology in therapeutic process. As such we did not distinguish between different types of technology. We referred to the rather general construct as online component with a therapeutic functionality; therefore, the effort expectancy was not included as an attribute. Social influence describes the degree to which individuals perceive that individuals or organizations whose opinions and views they appreciate believe that they should use a certain technology. Anticipating peer effects, we included social influence as the *recommendation* attribute, relating to both recommendations by colleagues and recommendations by professional society. Last, facilitating conditions are defined as organizational or technical conditions that encourage technology use [20]. We translated this determinant as a *reimbursement* attribute, which includes a financial incentive for therapists that could encourage the adoption of BC [23]. Because an important adoption factor identified in previous research was the presence of a good therapeutic relationship, which depends on the concrete operationalization of the blended treatment—the number of face-to-face sessions and amount of independent work of the client with the online component—we included the attribute *ratio* of online and personal sessions in our design [13, 15, 16]. The levels corresponding to the attributes were chosen not only to reflect realistic scenarios but also to provide a spread enabling respondents to differentiate between levels. In the third step, we conducted semi-structured interviews with therapists with different specializations to refine our selection of attributes and levels. Our aim was to represent all four main therapeutic schools and include therapists working in both inpatient and outpatient setting. Therefore, our interview sample consisted of two CBT (inpatient care), one psychodynamic therapist (outpatient care), and two humanistically oriented psychotherapists (outpatient care). We were not able to identify a therapist with systemic background who would be available for an interview. We stopped this qualitative phase of our study after these five interviews due to diminishing further information obtained regarding our experimental set-up and difficulties identifying additional interviewees. The final experimental design included five attributes with two to three levels each (see Table 1).

**Table 1:** Description of attributes and levels

Attribute	Level	Description
1) Recommendation	From a colleague; from a professional association; none	This feature refers to a recommendation that you received for the online component (tool or therapy program).
2) Proven effectiveness	9 out of 10 clients; 8 out of 10 clients; 7 out of 10 clients	This attribute describes the clinical effectiveness of the online component in comparison to no therapy based on the first studies, e.g., in the form “For 9 out of 10 clients, the online component (tool or program) was clinically effective”. Since 100% effectiveness has not yet been proven, the following options can be chosen: 7 out of 10, 8 out of 10, and 9 out of 10 clients.
3) Time ratio of face-to-face and online sessions	20:80; 50:50; 80:20	This attribute relates to the time invested and describes the percentage (%) ratio in which personal sessions and an online component (tool or program) are combined in an individual therapy process for each client. Examples are 20:80, 50:50 or 80:20, where the first number reflects face-to-face sessions and the second reflects the client’s independent work with an online component.
4) Reimbursement for the use of an online component	Proportional to time investment; proportional to time investment + lump sum	This attribute describes the reimbursement model for the use of BC: Proportional to the time invested for the online component per therapy block (preparation, follow-up work, supervision of homework, etc.) or rather proportional to the time invested for the application of an online component plus a lump sum.

### Choice tasks and experimental design

We constructed the choice tasks using unlabeled, paired comparisons. We used a fractional factorial design to reduce the response burden for participants [24]. A D-efficient Bayesian design was generated using JMP software from the SAS Institute. The design was optimized for main effects and all one-way interactions based on a conditional logit model. Attributes and levels were dummy coded, and Bayesian priors were obtained from a pretest. As the number of parameters to be estimated (main effects plus interaction effects) was larger than the maximum number of choice tasks we expected to still be feasible for respondents, the final design included 32 choice tasks that were divided into two blocks to guarantee response efficiency while facilitating robust statistical analysis. Participants were randomly allocated to one of the blocks of 16 choice tasks. An opt-out option was excluded, as our research question does not aim to predict likely adoption of a concrete online-tool or an eMHI as a part of BC but rather primary aims to estimate marginal rates of substitution among attributes, compare levels and attributes of the DCE. In this case, forced choice tasks are recommended. Furthermore, the exclusion of the opt-out alternative increases in order to increase the amount of information collected and avoid decreases interpretation bias [25].

### Survey design

The survey itself started by informing respondents about the definition of BC and the aim of the study. In addition to collecting sociodemographic information, the first part of the survey consisted of questions on attitudes towards and previous experience with eMHIs, as well as preferences for individual components of eMHIs, such as videoconferencing or CBT-based exercises. The second part started by familiarizing respondents with the DCE elicitation format and the types of questions that would be asked, followed by an unrelated warm-up choice task. Next, all attributes and levels of the DCE were explained in a narrative fashion before respondents had to answer the 16 choice tasks. Following the DCE, participants were asked about their general views of the advantages and disadvantages regarding BC and asked to evaluate the difficulty of DCE and the survey in general.

### Data collection

We administered the survey online through a market research agency specializing in health care providers (DocCheck Community GmBH) to individuals who, at that time, were working as psychotherapists in Germany. Data collection occurred in August 2020. Based on a rule-of-thumb calculation proposed by Johnson and Orme [26], the minimum required sample size for identifying the main effects in the choice experiment was 47 (or 140 for all two-way interactions). A final sample of 200 respondents was targeted to also provide sufficient power to investigate preference heterogeneity and allow for sufficient variation and therefore generalizability in terms of the experiences and expectations with and towards blended care.. We collected explicit and informed consent from respondents after providing them with a detailed explanation of how their personal data would be used. Each participating therapist received € 20 as monetary compensation from the market research agency.

Data collection was paused after 30 respondents. A pretest of the DCE was conducted among 30 therapists from the same respondent pool as in the main data collection. Data from the pretest were used to refine the questionnaire part of the survey, i.e., reducing the response burden [27], and to obtain the priors for the Bayesian D-efficient design (usually calculated using 10% of the sample). Data from this pretest was also used in the main analysis.

### Statistical analysis

Discrete choice data are commonly analyzed based on the random-utility framework [28] by applying different statistical models, which must be selected to fit the purpose of the study [29, 30]. In our case, attempting to elicit preferences for BC interventions among psychotherapists, who constitute a heterogeneous group concerning treatment styles or therapeutic focus, we expected a large variety of preferences. Therefore, to specifically model preference heterogeneity while also relaxing the independence of irrelevant alternative assumptions, a mixed logit model was estimated [31]. To select the utility function, the following steps were taken: First, we tested whether the inclusion of an alternative specific constant (ASC) and the inclusion of block dummy variables, indicating the survey version, would be necessary to obtain unbiased estimates based on a main effects mixed logit model [29]. Second, different functional forms of the effectiveness and face-to-face online ratio attributes were specified, namely, linear and logarithmic instead of categorical. Third, we tested several two-way interactions between attributes suspected to be correlated. Categorical variables were dummy coded throughout the analysis. In the final mixed logit model, 500 Halton draws were specified, all parameters for which we found heterogeneity were set to be random and normally distributed, and individual-level clustered standard errors were used. Marginal effects were calculated using the *mixlpred* command.

To investigate whether preferences specifically differed for various subgroups, interaction terms of the respective subgroup indicators (e.g., therapeutic style, clinic or outpatient, potential user of BC, previous user of BC, age, gender) and the main effects parameters were included in separate models. The significance of the difference in parameter estimates between subgroups was tested using *χ*^2^ tests for joint significance. We conducted two proposed tests to assess the internal validity of the choice experiment data [32]. First, using the *respdiff* command, we investigated the extent to which straight lining occurred, i.e., respondents always choosing either option A or option B, indicating a lack of serious engagement with the experiment. Second, attribute dominance, relating to noncompensatory preferences, was examined by calculating lexicography scores and counting the proportion of choices based on one attribute. We assumed attribute dominance if the lexicographic score was above 90%, as suggested previously [33]. All calculations were conducted using Stata 16 (StataCorp, College Station, TX).

## Results

### Respondent characteristics

The survey reached 1335 psychotherapists via e-mail who were members of the DocCheck Panel. A total of 238 respondents started the survey, 38 did not complete the questionnaire, and only three dropouts occurred after starting the DCE part of the survey. Table 2 shows the respondent characteristics as well as their experiences and expectations regarding BC formats. The age and gender distribution in the sample was similar to what has been reported for the overall population of psychotherapists in Germany (49%), while our sample likely was slightly younger [34]. We observed a realistic spread of therapeutic orientations across the main therapeutic approaches, although systemic and humanistic approaches were likely underrepresented^2^ [35]. Almost all respondents were medical psychotherapists practicing in inpatient facilities, while one-third worked predominantly in outpatient care.

**Table 2:** Sample characteristics and experiences with and expectations for BC

Respondent characteristics (n = 200)		Experiences and expectations regarding BC	
Mean age in years	48	**Experience with BC format in therapy**	
Female	43%	Yes	26.5%
**Therapeutic orientation**		No	73.5%
Behavioral	52.5%	**Evaluation of previous experience with BC**	
Psychodynamic or analytic	39%	Excellent	7.5%
Behavioral and psychodynamic or analytic	3%	Satisfied	54.7%
Systemic	1%	Neither good nor bad	32.1%
Humanistic	0.5%	Bad	5.7%
Another	4%	Very bad	0%
**Professional background**		**Willing to use BC in the future**	
Psychological psychotherapist	1.5%	Yes	90.5%
Medical psychotherapist	89%	No	8.5%
Child and adolescent psychotherapist	1%	**Preferred timing of BC application**	
Alternative practitioner for psychotherapy	0%	Stepped care before in-person treatment	9%
Psychiatrist	3.5%	Integrated parallel BC	68.5%
Psychiatrist and psychotherapist	3.5%	After in-person treatment	22.5%
General practitioner	1%	**Perceived main advantage of BC**	
Neurologist	0.5%	Time savings for therapists and patients	22.2%
**Main place of work**		Patient empowerment	21.3%
Own outpatient practice	33%	Increase in treatment efficacy	15%
Clinic/hospital	55%	Flexibility for therapists and patients	7.5%
Other	12%	Larger patient group can be reached	6.7%
**Satisfaction with monthly income**		Bridging waiting times for therapy	4.6%
Highly satisfied	8%	**Perceived main risk of BC**	
Satisfied	60.5%	Lack of personal support for patient	26.1%
Neither satisfied nor dissatisfied	20%	Deterioration of therapeutic alliance	17.4%
Dissatisfied	10%	Misinterpretation and treatment errors	23.1%
Very dissatisfied	1.5%	Overburdening patient compliance	9.3%
		Privacy risks	7.7%
		Low level of customization	6.7%
		Lack of therapeutic effectiveness	5.6%

### Experiences with and expectations for BC

**Fig. 1:**
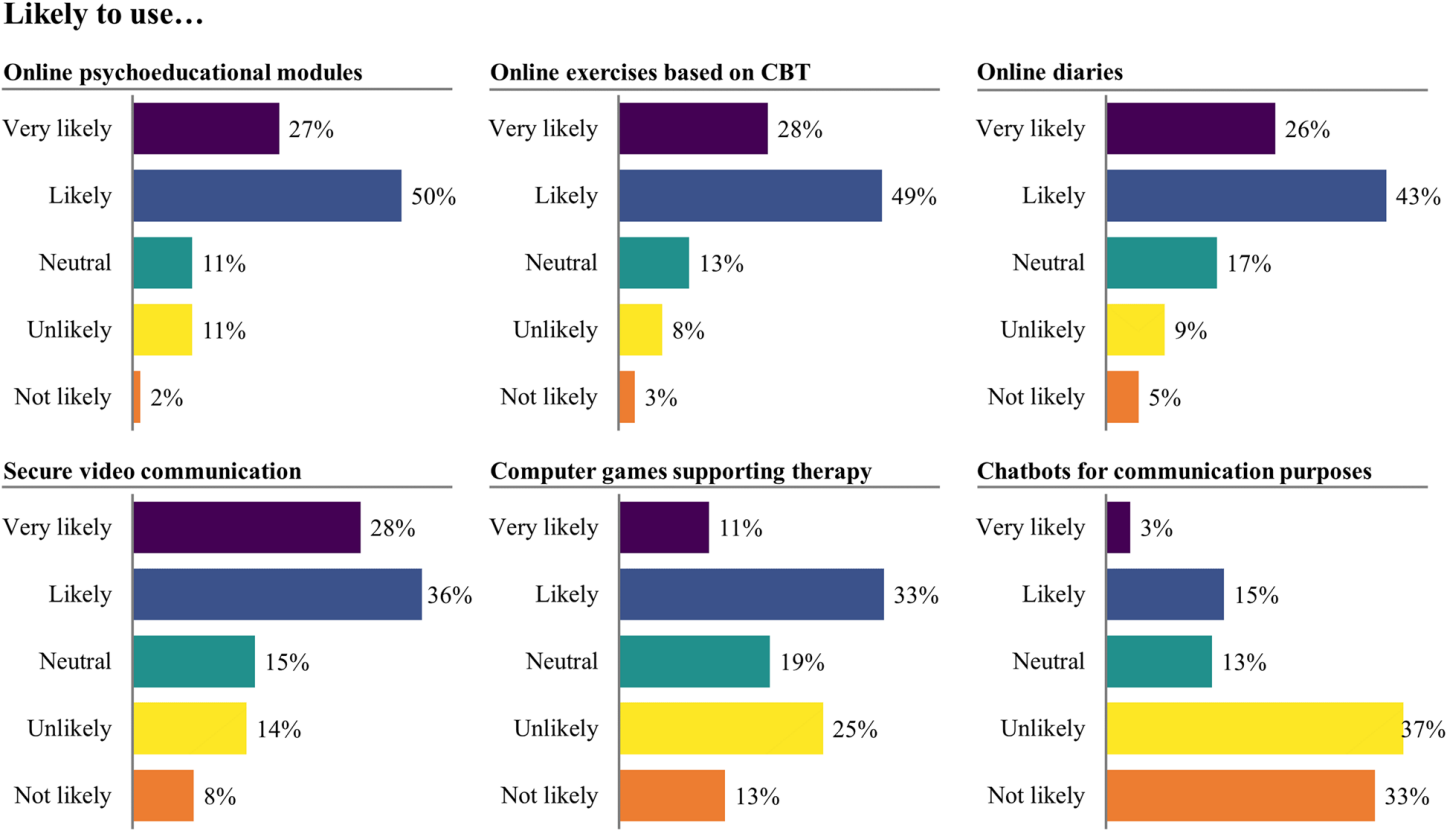
Likelihood of using BC features

Most respondents who had used BC before (26.5%) evaluated their experiences as positive (Table 2). Psychotherapists seemed willing to use BC in the future, mainly by integrating BC into regular therapy. The main reported reasons for not opting for BC in the future were the “too impersonal” character of treatment (55.6%), doubts regarding their effectiveness (11.1%), lack of compatibility with the performed therapy form (11.1%), and a lack of interest or need (5.6%). Time savings and patient empowerment were mentioned most often as potential advantages of BC, whereas lack of personal support and deterioration in the therapeutic alliance were seen as main risks. However, most respondents rated the likely impact of BC on the therapeutic alliance as positive (47.5%) or neutral (45.5%). The BC features most likely to be used in the future (Fig. 1) were psychoeducation, online exercises, online diaries, and secure video communication, while chatbots for communication were least likely to be used.

### Discrete choice experiment results

The small drop-out rate during the DCE (3 out of 203) and the type of qualitative comments collected on the overall survey indicated that the respondents were able to understand the choice tasks. Tests for the internal validity of the experimental data showed that straight lining occurred in four instances (2% of the sample), and non-compensatory behavior, with a lexicographic score of over 90%, was observed in 16% of the sample. These values lie close to the respective medians that were reported for these tests in a study summarizing 55 choice experiments [32]. In addition, 30 of these 32 observations exhibit dominant choice behavior for the ratio of online and personal sessions attribute. It is conceivable that for some psychotherapists, this attribute is indeed dominant and that non-compensatory behavior thus does not (always) imply decision heuristics.

The estimated utility function included an ASC and block-specific dummy variables to account for potential bias due to the positioning of the alternatives and the allocated survey version. Linear specifications of the effectiveness and face-to-face vs. online attributes were selected since the assumption of linear preferences regarding these two variables could not be rejected in models estimated only with categorical variables (*χ*^2^=0.02, p = 0.89 and *χ*^2^=1.46, p = 0.23, respectively). We tested the inclusion of two-way interactions between the additional reimbursement attribute and both the face-to-face vs. online attribute and the effectiveness attribute. Both were nonsignificant and therefore excluded in the final model. Therefore, for ease of interpretation, we refrained from including the interaction in the final model.

Table 3 contains the results of the mixed logit model. All main effects coefficients were significantly different from zero (columns 2 and 3), indicating their importance in the choice context. The signs of the attribute levels pointed in the expected direction, and the order of the recommendation attribute levels was logical (i.e., higher preference for recommendation by society than by colleagues), providing some confidence in the theoretical validity of the results. The recommendation by professional societies to use a certain BC intervention was the most important attribute level, while additional reimbursement played a minor role. Greater effectiveness and a higher face-to-face ratio of the BC intervention increased the likelihood of selecting this intervention. Preference heterogeneity was found for all included attributes, as indicated by the significant standard deviations of the parameters (columns 4 and 5). The marginal effects in column 6 demonstrate the change in likelihood of choosing a certain BC intervention if the attribute level changes compared to the reference category (or a unit change for linear variables), conditioning on all other attributes remaining constant. This also allows for a straightforward interpretation of the relative magnitude of the coefficients. Compared to no recommendation, a recommendation by a professional society increases the conditional likelihood of selecting a BC intervention by 25.7%. Using the effectiveness levels from the DCE, a change in effectiveness from 7 out of 10 to 9 out of 10 increased the choice likelihood by a slightly lower factor (21.0%). The smallest marginal effect was found for the reimbursement attribute (9.5%).

**Table 3:** DCE results based on a mixed logit model

	Preference estimates				Marginal effect
Attributes and levels	Coefficient	95% CI	SD	95% CI of SD	
Recommendation					
None	Reference				Reference
Colleagues	1.30	[0.94,1.65]	1.46	[0.97,1.96]	12.2%
Professional societies	2.70	[2.12,3.27]	2.45	[1.78,3.11]	25.7%
Effectiveness (linear)	1.08	[0.85,1.30]	1.02	[0.73,1.30]	
8 of 10 vs. 7 of 10					10.7%
9 of 10 vs. 7 of 10					21.0%
Face to face vs. online	0.03	[0.01,0.04]	0.09	[0.07,0.11]	
50:50 vs. 20:80					7.4%
80:20 vs. 20:80					14.2%
Reimbursement					
Proportional to time	Reference				Reference
Time + lump sum	0.95	[0.66,1.23]	1.33	[1.02,1.63]	9.5%
ASC	−0.32	[−1.33,0.69]	1.37	[0.59,2.15]	
ASC x block2	0.05	[−0.97,1.08]	1.39	[0.51,2.27]	
ASC x block3	0.29	[−0.70,1.29]	1.63	[0.58,2.67]	
Log likelihood	−1568				
AIC	3223				
BIC	3521				
Respondents	200				
Observations	6400				

*Note*. Attributes were dummy coded. Coefficients refer to the mean preference estimates and standard deviations (SD) of the distribution around the means. Uncertainty around the mean and SDs is shown using 95% confidence intervals (CIs)

**Fig. 2:**
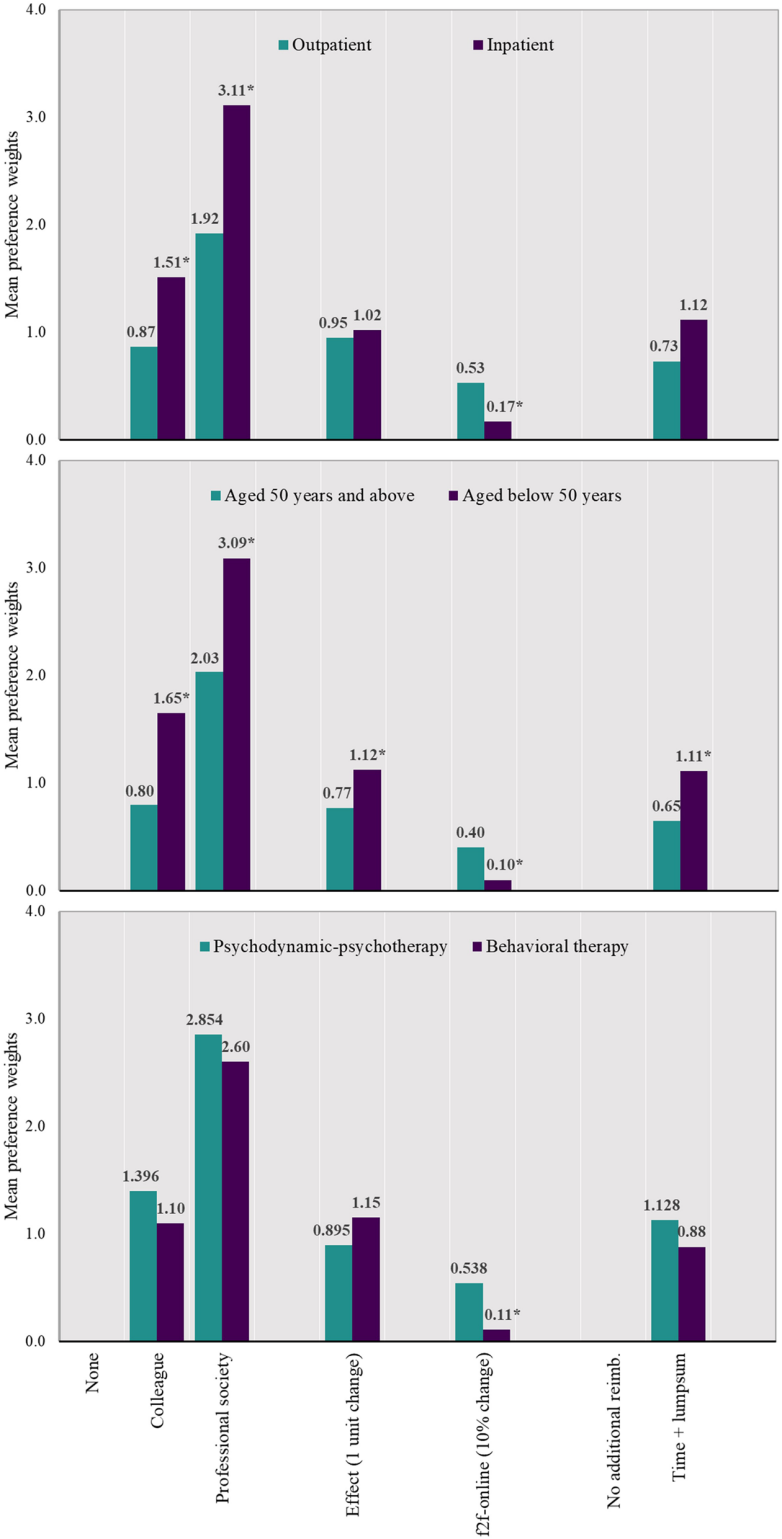
Subgroup results for age, outpatient vs. inpatient setting, and type of therapy. Note. Interaction term significant at the 10% level. Abbreviation: f2f-online, ratio of face-to-face vs. online time

In terms of the subgroups, we found significantly different coefficient estimates for psychotherapists working in the inpatient vs. outpatient setting (*χ*^2^=15.70, *p* < 0.01), for respondents being younger or older than 50 years (*χ*^2^=17.07, *p* < 0.01), and for therapists predominantly practicing behavioral therapy vs. psychodynamic psychotherapy (*χ*^2^=16.62, *p* < 0.01). Figure 2 presents the coefficient estimates for the respective subgroups. Younger therapists put more weight on recommendations to use a BC format, the potential effectiveness of the intervention and additional reimbursement, while a higher share of face-to-face vs. online time was less important to them. Inpatient therapists are more influenced by recommendations than are outpatient therapists, who preferred a higher share of face-to-face vs. online time. Face-to-face time was significantly less important for therapists predominantly conducting behavioral therapy than for therapists conducting psychodynamic psychotherapy.

In a deviation from the main model specification we tested the inclusion of an interaction between the linear effectiveness and face-to-face vs. online attributes (see Additional file 1 Appendix Table A1). The interaction between face-to-face time and effectiveness was positive, meaning that the utility of an option with higher effectiveness and higher face-to-face time did not just increase by the additive effect of effectiveness and face-to-face time, but that the combined effect would be even larger. In other words, therapists value a higher effectiveness even more if it coincides with more f2f-time and vice versa. Finding that the coefficients of face-to-face time were not significant anymore after the inclusion of the interaction is hinting towards that face-to-face time isolated may not have much value on its own if effectiveness is low. Preference estimates for the remaining attributes were largely unaffected by the inclusion of the interaction term.

## Discussion

Conducting a survey, including a DCE, with a sample of 200 psychotherapists from Germany, our study assessed psychotherapists’ experiences, expectations and preferences regarding BC psychotherapy formats, which combine the application of eMHIs or online-tools and regular psychotherapy. The study’s main contributions are as follows. First, to our knowledge, this is the first DCE exploring preferences among psychotherapists for BC. Second, our survey described and distinguished among concrete BC characteristics and application scenarios, making our analysis as tangible as possible for psychotherapists. Third, we defined BC as an application of an online component with a therapeutic functionality, explicitly excluding the use of communication technologies for therapeutic purposes, a distinction that usually was not being clearly made in previous research [9, 13–16]. Fourth, this study is the first survey, and DCE, that has investigated preferences for BC in the context of Germany, an example of a country with low e-mental health utilization.

In general, we found rather positive attitudes towards BC among German psychotherapists and a strong willingness to use BC in the future. This finding was surprising, as previous studies on the attitudes of German-speaking psychotherapists towards eMHIs and BC indicated that they had reservations [15, 17]. This positive attitude might be explained by the timing of our study that was conducted during the COVID-19 pandemic. The outbreak of the pandemic was called a “turning point” for e-mental health and pushed the utilisation technologies for therapeutic purposes worldwide [1]. Positive attitudes towards BC are however in concordance with favorable attitudes of Dutch therapists [13, 14, 16]. Most therapists prefer using BC features such as psychoeducation, online exercises, online diaries, and secure video communication integrated into regular therapeutic cycles instead of pre- or post-therapy applications. Similar results were found in a Delphi study in which Dutch therapists found practical therapy components such as assignments, diaries and psychoeducation most suitable for a digital format [13]. This preference was also found in other studies among therapists from the Netherlands and Austria [15, 16]. Most respondents perceived a positive or neutral impact of BC on the therapeutic relationship. It is not surprising that therapists conducting psychodynamic therapy are less likely to use BC treatments with a large online component than behavioral therapists, as BC is mainly grounded in CBT, which was also found in other studies [14–17]. The main reasons for not applying BC were lack of personal contact, doubts regarding effectiveness and lack of personal interest. These findings are in concordance with previous research across different countries, where fears of losing contact with patients, not offering patients the amount of help they need, or making treatment mistakes were frequently expressed [13–15, 18]. A previous choice experiment from Germany among the general population also showed that accompanying in-person contact is perceived as a central need when using eMHIs [10]. The main stated advantages of BC among therapists coincide with the first findings referring to time savings, patient empowerment expressed in more independent patient-therapist relationship, and the perception of increased treatment effectiveness, which was also shown in other studies [13, 14, 36]. The preference results showed that a recommendation to use a BC treatment format made by a professional society (psychotherapist association) was most influential when choosing between the treatment scenarios in the choice experiment. The effect was even larger among therapists working in an inpatient setting. A higher effectiveness was considered important and to a lesser extent also a larger share of face-to-face vs. online time. The latter, however, appears to be rather dependent on a sufficient level of effectiveness and may not be very valuable to therapists in itself. Additional reimbursement for BC was found to be less important, especially among older providers. This financial incentive does not appear to be important for German therapists. In previous research, cost-effectiveness or cost reduction were identified to be relevant in this context [12, 36]. While these potentially only indirectly financially benefit therapists, this is somewhat at odds with our findings. The results of our experiment show that financial dimension doesn’t play a major role for German psychotherapists. This is an interesting finding that indicates the presence of different attitudes and values of health care actors and providers participating in the current digital health discourse that need to be explored in further research. Finally, we acknowledge that the definition of BC we used in our survey may affect the generalizability of our results. Our recommendation for the future research is to make the definition of BC as clear as possible during the research process towards participants as well as on the later stage while reporting.

### Limitations

Although our study was carefully designed and tests for internal validity revealed satisfactory results, we need to acknowledge the following research limitations related to the external validity of our findings. In Germany, both qualified psychologists *and* physicians (including psychiatrists), who have completed several years of specialist practical training and certification in psychotherapy, are authorized to practice psychotherapy. While providing us direct access to a sufficiently large pool of psychotherapists, the selected sampling agency (DocCheck) has a drawback in that most of the panel’s members have a medical background, resulting in our sample mainly consisting of physicians with psychotherapeutic training (89%). The share of medical psychotherapists among office-based Statutory Health Insurance psychotherapists was 24% in 2017. However, this represents only one portion of all ambulatory psychotherapy providers. This share is likely to be higher in outpatient departments of psychiatric and psychosomatic facilities, which also provide ambulatory psychotherapy and are likely users of eMHIs. However, data about the educational background of all therapists providing ambulatory psychotherapy in Germany are not available. Thus, we estimate that the share of medical psychotherapists among all therapists providing ambulatory psychotherapy in Germany is lower in the total population than in our sample.

A further limitation relates to the selected characteristics in the choice experiment. While these were carefully selected using standard practices, including interviews with providers, the experimental design could have omitted important characteristics, in part because no choice experiments have been conducted in this novel but emerging research area. That BC treatment formats are also new to many psychotherapists, who often have little to no prior experience, may also be seen as a limitation. While the DCE allows us to present hypothetical scenarios, the structure, mechanism, and potential benefits/risks of BC thus remained quite abstract to respondents.

Finally, our study was conducted during the COVID-19 pandemic, where measures such as contact restrictions increased the necessity to use digital technology in mental health care. In light of this, the attitudes towards BC among therapists might have been more positive compared to pre-pandemic levels. Furthermore, it is unclear to what extent the obtained preferences will remain stable after the pandemic.

## Conclusion

German medical psychotherapists, despite having little previous experience with BC, showed positive attitudes towards BC together with a strong intention for future use in treatments. Similar to therapists from other countries, they appreciate the use of eMHIs for practical CBT-oriented therapy tasks while stressing the importance of maintenance of the therapeutic relationship through the larger number of parallel face-to-face sessions. Our findings from the DCE suggest a strong preference for BC treatment that includes an online component approved by a professional psychotherapist society. This highlights the importance of including professional associations early in the development, application, and evaluation of BC treatments to encourage uptake. Our results suggest that German psychotherapists care less about the additional reimbursement but are ready to use BC formats if they are convinced of the effectiveness and trustworthiness of the online components. Thus, financial incentives may not be very useful for encouraging wider use of BC in Germany, and assessment along with recommendations from trusted institutions for online components of BC would be recommended.

## Supplementary Information

**Additional file 1.**

**Additional file 2.**

**Additional file 3.**

## Data Availability

The data-sets generated and/or analysed during the current study are available in the following repository “Preferences for e-Mental Health Interventions in Germany: A Discrete Choice Experiment”, available at https://osf.io/af2rw/?view_only=e148be5df318487486bd2a406369abf1

## Abbreviations

AIC: Akaike’s Information Criteria
ASC: Alternative specific constant
BC: Blended Care
BIC: Bayesian Information Criteria
CBT: Cognitive Behavior Therapy
CI: Confidence Interval
COVID-19: Coronavirus Disease 2019
DCE: Discrete Choice Experiment
eMHIs: Online- or Web-based Therapeutic Interventions
JMP software: Statistical Software
ISPOR: International Society for Pharmacoeconomics and Outcomes Research
SD: Standard Deviations
UTAUT: Unified Theory of Acceptance and Use of Technology

## Footnotes

1 France, Germany, Netherlands, Poland, Spain, Sweden, Switzerland, and the United Kingdom

2 As psychoanalysis, psychodynamic therapy, and cognitive behavioral therapy meet the requirements of German health insurance companies, mental health professionals usually opt for one of these three specializations in their postgraduate training.
