# Supplementary material for: Preferences of psychotherapists for blended care in Germany: a discrete choice experiment
Source: BMC Psychiatry. 2022 Feb 12;22:112. doi: 10.1186/s12888-022-03765-x (PMC8841060; doi:10.1186/s12888-022-03765-x)
Supplement: Supplementary file 1 — Additional file 1. [file 12888_2022_3765_MOESM1_ESM.docx]

**Appendix**

Table A1: DCE results based on mixed logit model including interaction

| Attributes and levels | Coefficient | 95% CI |
| --- | --- | --- |
| Recommendation |  |  |
| None | Reference |  |
| Colleagues | 1.29 | [0.95,1.63] |
| Professional societies | 2.76 | [2.21,3.31] |
|  |  |  |
| Effectiveness (linear) | 0.74 | [0.43,1.04] |
| 8 of 10 vs. 7 of 10 |  |  |
| 9 of 10 vs. 7 of 10 |  |  |
|  |  |  |
| Face to face vs. online | -0.02 | [-0.06,0.03] |
| 50:50 vs. 20:80 |  |  |
| 80:20 vs. 20:80 |  |  |
| I (Effectiveness X f2f-time) | 0.006 | [0.001-0.010] |
|  |  |  |
| Reimbursement |  |  |
| Proportional to time | Reference |  |
| Time + lump sum | 0.93 | [0.67,1.19] |
|  |  |  |
| ASC | -0.19 | [-0.69,0.30] |
| ASC x block2 | -0.08 | [-0.62,0.45] |
| ASC x block3 | 0.26 | [-0.31,0.82] |
| Log likelihood | -1,600 |  |
| AIC | 3,238 |  |
| BIC | 3,359 |  |
| Respondents | 200 |  |
| Observations | 6,400 |  |

Note: I (Effectiveness X f2f-time) is an interaction effect between the linear effectiveness and f2f-time variables.
