# Supplementary material for: Preferences of psychotherapists for blended care in Germany: a discrete choice experiment
Source: BMC Psychiatry. 2022 Feb 12;22:112. doi: 10.1186/s12888-022-03765-x (PMC8841060; doi:10.1186/s12888-022-03765-x)
Supplement: Supplementary file 2 — Additional file 2. [file 12888_2022_3765_MOESM2_ESM.docx]

**Summary of responses of the qualitative interviews**

1. Three of five interview partners did not have a practical experience with blended care. The experiences of others were limited to the psychoeducation context.
2. All interview partners expressed the need in additional information and education about e-mental health and application formats of blended care. The participation of professional association in promotion was considered as important by all interview partners.
3. The chances of BC were seen in more time resources and more patients for therapists, potential cost-effectiveness as well as easier access to mental health care for potential patients. The interview partners had difficulties to indicate tangible risks regarding BC.
4. All interview partners indicated as very and equally important the following themes: independent certification for BC digital solutions and external evaluation for BC solutions regarding their clinical effectiveness, technical efficiency, ease of use, ethical harmlessness and cost-effectiveness.
5. Digital solutions for psychoeducation modules, CBT skills training, progress monitoring, online diaries, secure video and online communication were considered as helpful while video games, virtual reality and chat bots were less known and were considered as less useful.
6. CBT therapists preferred extended independent work of clients with digital component, while other therapists stressed the importance of personal contact (less digital format, more face-to-face sessions).
7. While CBT therapists were positive regarding influence of BC on the therapeutic relationship, other therapists find it difficult to assess the effects of BC on the therapeutic relationship and indicated that further research on this topic is needed.
