# Supplementary material for: Preferences of psychotherapists for blended care in Germany: a discrete choice experiment
Source: BMC Psychiatry. 2022 Feb 12;22:112. doi: 10.1186/s12888-022-03765-x (PMC8841060; doi:10.1186/s12888-022-03765-x)
Supplement: Supplementary file 3 — Additional file 3. [file 12888_2022_3765_MOESM3_ESM.docx]

Supplemental material

**Main analysis specification run with 30 respondents from pre-test:**

|  | | Preference estimates | | | | |  | | |  |
| --- | --- | --- | --- | --- | --- | --- | --- | --- | --- | --- |
| Attributes and levels | Coefficient | | 95% CI | SD | 95% CI of SD | | |  | | |
| **Recommendation** |  | |  |  |  | |  | | |  |
| None | Reference | |  |  |  |  | | |  |  |
| Colleagues | 0.002 | | [-0.86,0.86] | 1.18 | [0.38,1.98] |  | | |  |  |
| Professional societies | 2.41 | | [-0.07,4.90] | 2.38 | [0.15,4.62] |  | | |  |  |
|  |  | |  |  |  |  | | |  |  |
| **Effectiveness (linear)** | 0.53 | | [0.13,0.93] | 0.78 | [0.08,1.49] |  | | |  |  |
|  |  | |  |  |  |  | | |  |  |
| **Face to face vs. online** (linear) | 0.07 | | [-0.01,0.14] | 0.10 | [0.02,0.18] |  | | |  |  |
| **Reimbursement** |  | |  |  |  |  | | |  |  |
| Proportional to time | Reference | |  |  |  |  | | |  |  |
| Time + lump sum | 0.41 | | [-0.11,0.94] | 0.65 | [-0.28,1.58] |  | | |  |  |
|  |  | |  |  |  |  | | |  |  |
| **ASC** | -0.76 | | [-1.44,-0.08] | 0.60 | [-0.15,1.35] |  | | |  |  |
| Log likelihood | -213.58 | |  |  |  |  | | |  |  |
| AIC | 451.2 | |  |  |  |  | | |  |  |
| BIC | 509.6 | |  |  |  |  | | |  |  |
| Respondents | 30 | |  |  |  |  | | |  |  |
| Observations | 960 | |  |  |  |  | | |  |  |
